# *In silico* genome wide mining of conserved and novel miRNAs in the brain and pineal gland of *Danio rerio* using small RNA sequencing data

**DOI:** 10.1016/j.gdata.2015.11.013

**Published:** 2015-11-26

**Authors:** Suyash Agarwal, Naresh Sahebrao Nagpure, Prachi Srivastava, Basdeo Kushwaha, Ravindra Kumar, Manmohan Pandey, Shreya Srivastava

**Affiliations:** a Division of Molecular Biology and Biotechnology, ICAR-National Bureau of Fish Genetic Resources, Lucknow 226 002, Uttar Pradesh, India; b AMITY Institute of Biotechnology, AMITY University Uttar Pradesh, Lucknow Campus, Lucknow 226 028, India

**Keywords:** MiRNA, NcRNAs, Novel miRs, Phylogenetic analysis, Zebrafish

## Abstract

MicroRNAs (miRNAs) are small, non-coding RNA molecules that bind to the mRNA of the target genes and regulate the expression of the gene at the post-transcriptional level. Zebrafish is an economically important freshwater fish species globally considered as a good predictive model for studying human diseases and development. The present study focused on uncovering known as well as novel miRNAs, target prediction of the novel miRNAs and the differential expression of the known miRNA using the small RNA sequencing data of the brain and pineal gland (dark and light treatments) obtained from NCBI SRA. A total of 165, 151 and 145 known zebrafish miRNAs were found in the brain, pineal gland (dark treatment) and pineal gland (light treatment), respectively. Chromosomes 4 and 5 of zebrafish reference assembly GRCz10 were found to contain maximum number of miR genes. The miR-181a and miR-182 were found to be highly expressed in terms of number of reads in the brain and pineal gland, respectively. Other ncRNAs, such as tRNA, rRNA and snoRNA, were curated against Rfam. Using GRCz10 as reference, the subsequent bioinformatic analyses identified 25, 19 and 9 novel miRNAs from the brain, pineal gland (dark treatment) and pineal gland (light treatment), respectively. Targets of the novel miRNAs were identified, based on sequence complementarity between miRNAs and mRNA, by searching for antisense hits in the 3′-UTR of reference RNA sequences of the zebrafish. The discovery of novel miRNAs and their targets in the zebrafish genome can be a valuable scientific resource for further functional studies not only in zebrafish but also in other economically important fishes.

## Graphical abstract

**Figure:**
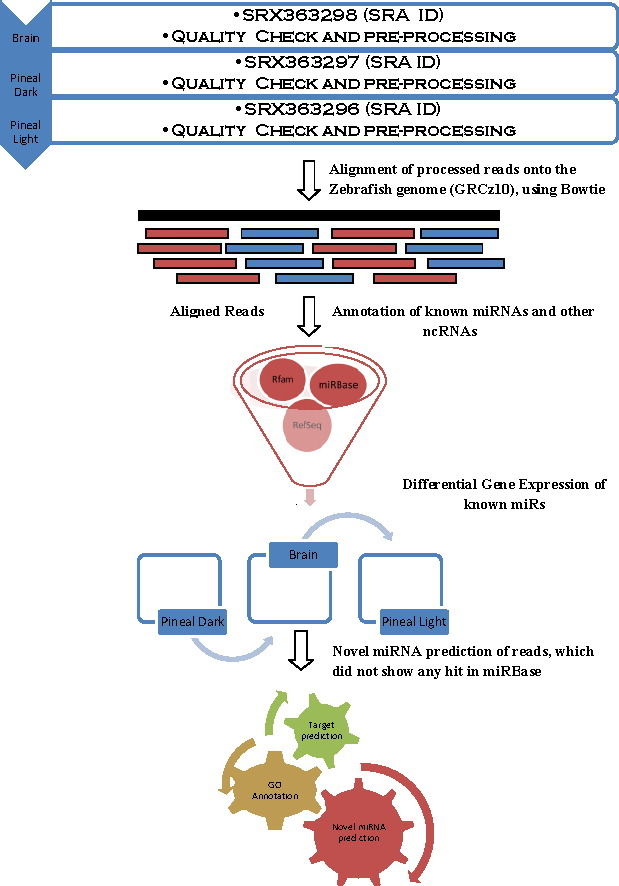

## 1 Introduction

The discovery of the first miRNAs in *Caenorhabditis elegans* in 1993 paved the way for unearthing of thousands of the mature miRNAs in a wide variety of organisms, including animals, plants and even viruses [1], [2], [3]. In recent years, intensive studies have been carried out on identification of novel miRNAs and their targets, with the addition of newly discovered miRNAs to miRBase and subsequently resulting in its new version release. The current miRBase release 21 has miR information on 9 fish species. MiRNAs are a distinct class of endogenous RNA molecules, which do not code for any protein and are about 22 nucleotides in length [4]. MicroRNAs (miRNAs) are small, non-coding RNA molecules that function as master regulators of the genome. They bind to the mRNA of the target genes; thus, regulating the gene expression at the post-transcriptional level. Many miR-related discoveries have come from zebrafish investigations [5], [6], [7], [8], [9], [10]. After the release of Zv9 (zebrafish genome draft), the zebrafish genome project joined the Genome Reference Consortium (GRC) for further improvement and ongoing maintenance. The GRC has now released a new reference assembly, GRCz10. Correlating the miR functions from a model organism to that of human health largely depends on recognizing true orthologs of human miRs. Thus, for the benefit of the entire miR community, a better understanding of the miRNome is essential [11].

Each miRNA appears to regulate the expression of tens to hundreds of genes to efficiently coordinate multiple cellular pathways. Precursor-miRNAs are usually 60–80 nucleotides in length with a hairpin secondary structure while mature miRNAs are mostly 18–26 nucleotides [12]. Many miRNAs are conserved across vertebrates [13]. Mutation in miRNA genes or the improper miRNA and target gene interaction may become a cause of various genetic diseases. With the advent of high-throughput sequencing technologies, non-conserved or weakly expressed miRNAs, along with species-specific miRNAs can be identified from a wide range of organisms [14], [15], [16], [17], [18]. Recent studies have focused on bioinformatic analysis of the NGS data obtained from small RNA sequencing, where algorithms predict miRNA precursor molecules based on the presence of hairpins and other associated parameters and how they are processed into mature miRNAs. This sort of analysis can lead to the discovery of both novel and evolutionary conserved miRNAs. Kloosterman et al. [19] reported 66 new miRNAs and 11 star sequences corresponding to 116 potential miRNA hairpins in the zebrafish genome by deep sequencing of two small RNA cDNA libraries. Bizuayehu et al. [20] worked on the Atlantic Halibut and the results indicate a wide conservation of miRNA precursors and involvement of miRNA in multiple regulatory pathways. Despite enormous research on zebrafish, the annotation of miR-producing genes remains limited.

Zebrafish is a fish species of freshwater ecosystem and considered popular organism for studying the gene functions of the vertebrate, especially human development and genetic diseases. It is a favored model organism due to their specific features such as virtually transparent embryos, small size, ability to keep them together in large numbers, ease of breeding and easy to maintain/manipulate/observe in the lab experiments. The critical role of miRNAs in gene expression is highly evident from the recent studies in zebrafish. The miRNAs play key roles in zebrafish organ formation and their expression at different time points.

In the present work, we used Illumina HiSeq2000 small RNA sequencing data from the brain and pineal gland (dark and light treatments) of zebrafish from NCBI SRA database. The total number of reads in the data obtained from NCBI SRA was ~ 6 million, ~ 10.4 million and ~ 14.8 million for the pineal light, pineal dark and brain, respectively. An integrative bioinformatic strategy was applied to detect and analyze the whole miRNA transcriptome of zebrafish. The present study led to the discovery of novel miRNAs in the brain and pineal gland of zebrafish, which will contribute for a better understanding of the role miRNAs play in regulating diverse biological processes.

## 2 Materials and methods

### 2.1 Raw data retrieval and pre-processing

The small RNA sequencing data from three mature miRNA libraries (pineal light, pineal dark and brain) of zebrafish were downloaded from NCBI SRA (*SRX363296*, *SRX363297* and *SRX363298*) and were subsequently used for analysis in the present study. The downloaded data was in SRA format, which was subsequently converted to fastq format using sratoolkit (version 2.3.4-2) [30], fastq-dump option. The obtained data was generated on HiSeq2000 using standard Illumina sequencing workflow with the multiplexing option. A custom Perl script was written to remove low quality bases, adaptor sequences, count the number of occurrences of each read, and eliminate reads outside the targeted size range (≥ 16 and ≤ 30).

### 2.2 Identification of conserved miRNAs and other ncRNAs in zebrafish and other fishes

The filtered reads were further aligned onto the latest released version of zebrafish genome GRCz10, using bowtie [31] with two mismatches and zero gaps. Only the aligned reads were used for the downstream analysis. MiRBase [32], [33] release 21 was used for annotation of known miRNA. A custom based Perl script was written in order to extract only the fish miRNA from the miRBase, along with the preparation of unique fish miRNA database, and its annotation files. Aligned reads were annotated against the unique fish miRNA database, RefSeq database, as well as noncoding RNA sequences of zebrafish from Rfam (version 11) [34]. A custom Perl script was written in order to extract the best read hit, which depicts the fish miRNA, and to segregate the miRNA hits into known zebrafish miRNA and other fish miRNA.

### 2.3 Differential expression of known miRNAs

The individual read counts of the 3 data sets were fed into a custom based Perl script to prepare the final read count table, which was taken as input for DeSeq [35]. The results were further segregated into up-regulated, down-regulated and neutral miRs based on the log2 fold change value. For the log2 fold change greater than 1, less than − 1 and between 1 and − 1, the miRs were designated as up, down and neutral miRs. A heatmap of few highly regulated miRNAs was drawn using R script.

### 2.4 Identification of novel miRNA candidates

The reads with no hits in the unique fish miRNA database and Rfam were further used for the prediction of putative new miRs using Mireap (version 0.2). All novel pre-miRNAs were identified based on the presence of a classic hairpin structure [36]. These filtered small RNA reads were aligned with the zebrafish genome using bowtie with strict parameters (number of mismatch; — v = 0). Mireap was used for the detection of novel miRs based on alignment, secondary structure, free energy and location on the precursor arm. The parameters used for Mireap prediction included: i) minimal miRNA length = 18; ii) maximal miRNA length = 26; iii) minimal miRNA reference length = 20; iv) maximal miRNA reference length = 24; v) uniqueness of miRNA = 20; vi) maximal energy allowed for a miRNA precursor = − 18 kcal/mol; vii) minimal and maximal space between the miRNA and miRNA* = 5 and 35 respectively; viii) minimal mature pair = 14; ix) maximal mature bulge = 4; x) maximal duplex asymmetry = 5; and xi) flank sequence length = 100 [37]. It is evident from the previous studies that more than 90% of miRNA precursors have MFEIs greater than 0.85 (tRNAs ~ 0.64, rRNAs ~ 0.59, and mRNAs ~ 0.65) [38]. Therefore, minimal folding free energy index (MFEI) is a new criterion for assaying miRNAs and distinguishing miRNAs from other non-coding and coding RNAs. MFEI is equal to MFE / (precursor length) × 100 / (G + C)%. In the present study, reads which showed MFEI value to be greater than 0.85, were considered as novel miRNAs candidates.

### 2.5 Target prediction of novel miRs

Target prediction of the novel miRNA was done against the 3′-UTR sequences of zebrafish (www.ensembl.org/biomart) using miRanda (version 3.3a) [39]. The miRanda results were parsed based on score > 150 and energy <− 20. The gene ontology (GO) terms of the predicted targets were taken from UniProt database and were further classified into most abundant GO terms for biological process, cellular component and molecular function.

## 3 Results and discussion

### 3.1 Raw data retrieval and pre-processing

**Fig. 1:**
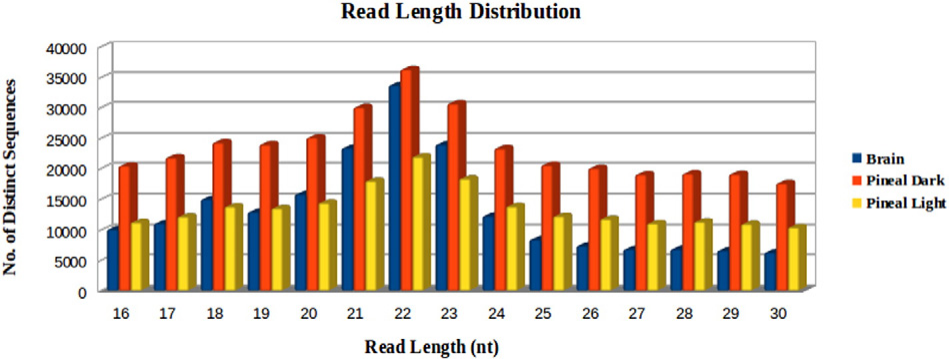
Read length distribution. The figure represents the read length distribution of the 3 data sets after length filtering.

NCBI SRA was the main source of NGS data, from where the small RNA sequencing data of three mature miRNA libraries (pineal light, pineal dark, and brain) of zebrafish (*SRX363296*, *SRX363297* and *SRX363298*) was downloaded and was subsequently used in the present study. The total numbers of reads were found to be ~ 6 million, ~ 10.4 million and ~ 14.8 million for the pineal light, pineal dark and brain, respectively. A custom Perl script was written to remove low quality sequences, adapter sequences and to count the number of occurrences of each read, with the elimination of reads outside the targeted size range (≥ 16 and ≤ 30). A read length distribution graph (Fig. 1) was prepared to get insight into the number of reads at each read length. The maximum numbers of distinct sequences were found to be of 22 bp and most of the data was falling between 21 and 23 bp in all the 3 data sets. The numbers of distinct sequences were calculated both before and after length filtering. Finally, the total numbers of distinct sequences after length filtering (Table 1), which were considered for downstream analyses were 200,520, 204,696 and 351,638 for the brain, pineal light and pineal dark, respectively.

### 3.2 Identification of conserved miRNAs and other ncRNAs in zebrafish and other fishes

The filtered reads were further aligned onto the latest released version of zebrafish genome GRCz10, using bowtie with two mismatches and zero gaps. A total of 88.58%, 60.00% and 61.14% reads aligned to the zebrafish genome GRCz10 for the brain, pineal gland (dark) and pineal gland (light), respectively. Only the aligned reads were used for the downstream analysis. miRBase release 21 was used to determine the known and conserved miRNAs in fishes. MiRNA data of 9 fishes was extracted from miRBase, comprising of 1637 miRNA sequences. Unique fish miRNA database was prepared by eliminating the redundant sequences, which comprised of 1029 sequences. The aligned reads from all the 3 data sets were blast against the unique fish miRNA database. A total of 165, 151 and 145 known zebrafish miRNAs were found in the brain, pineal gland (dark treatment) and pineal gland (light treatment) along with their expression values and were further annotated for their presence in other fishes (Table S1). A total of 221, 196 and 195 distinct reads showed hits with other fish miRNAs in the brain, pineal gland (dark treatment) and pineal gland (light treatment), respectively (Table S1).

**Fig. 2:**
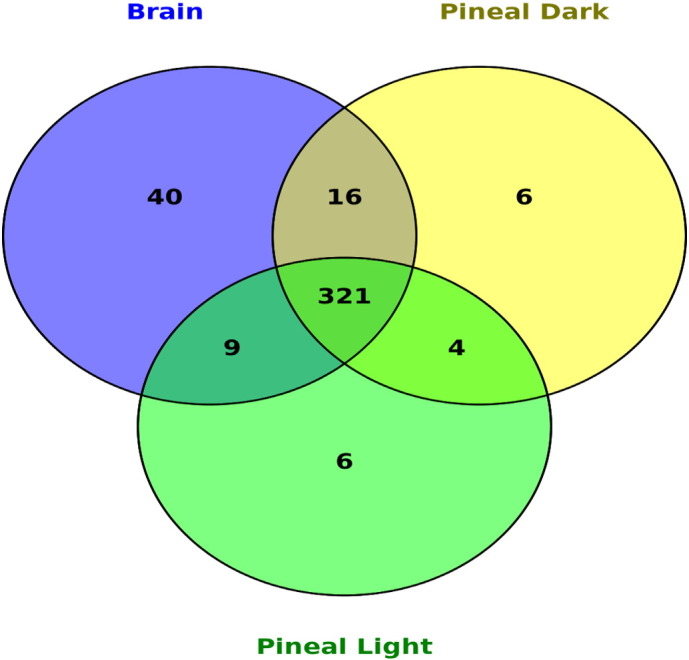
Venn diagram of conserved miRNAs in the brain, pineal gland (dark treatment) and pineal gland (light treatment).

A comparative study of all the detected fish miRNAs showed 321 miRNAs to be common along all the 3 data sets, with 40, 6 and 6 miR genes to be uniquely expressed in the brain, pineal gland (dark treatment) and pineal gland (light treatment), respectively (Fig. 2). The maximum read count was observed for miR-181a and miR-182 in the brain and pineal gland, respectively. The miR-181a is mainly involved in proliferation, and is an active miRNA regulating regeneration process. Rudnicki et al. [21] and Frucht et al. [22] also reported that miR-181a can encourage proliferation in both quiescent and proliferating chick basilar papilla. It is also reported that over-expression of miR-182 results in production of ectopic hair cells [21]. The miR-182 forms a part of miR-183/96/182 cluster, whose expression is considerably enriched in the pineal gland and up-regulated by light [23], [24]. Because of the role of these miRNA in promoting regeneration and mediating the targets and transcriptional factors involved in regeneration, these miRNAs may prove to be promising in therapeutics.

**Fig. 3:**
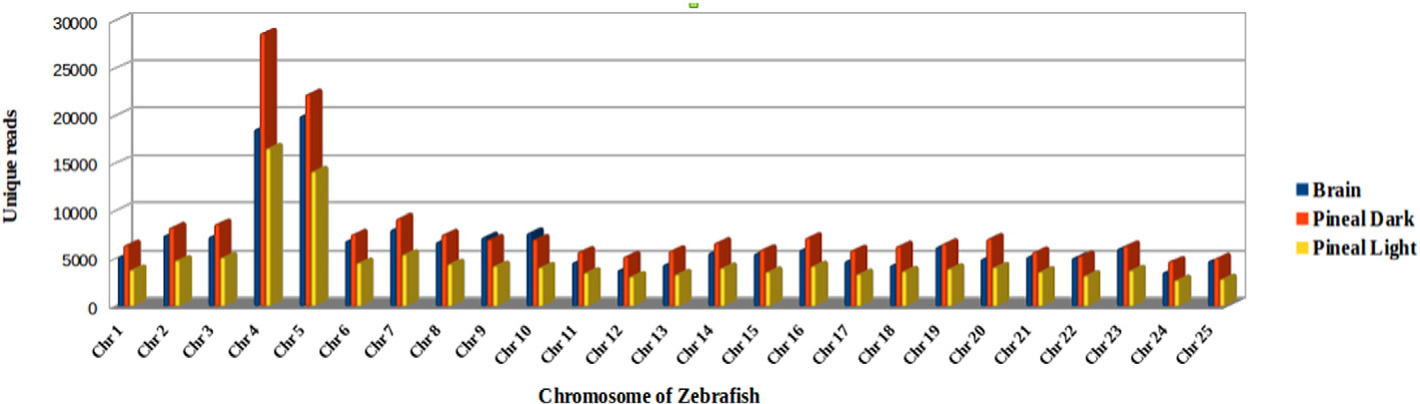
Distribution of unique miRNA reads across the zebrafish genome.

**Fig. 4:**
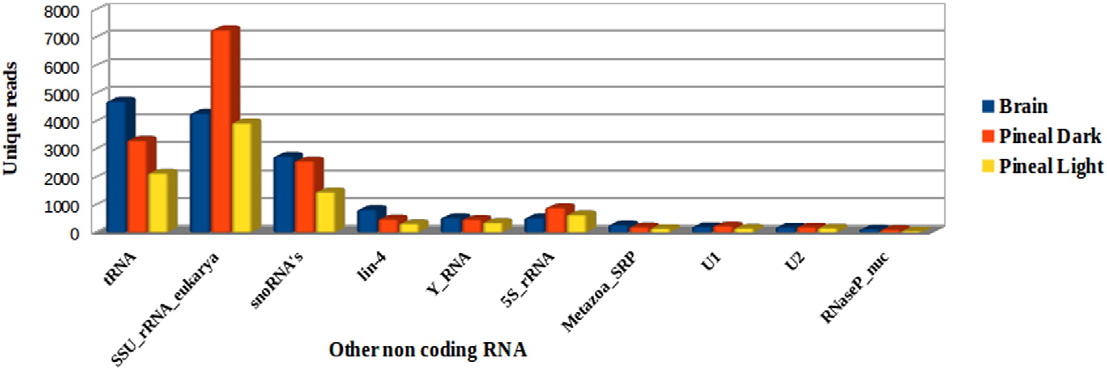
Unique reads representing other ncRNAs in the data.

Chromosome wise coverage of the reads aligning to zebrafish genome GRCz10 showed that the maximum numbers of miR genes were coded by chromosomes 4 and 5 (Fig. 3). Thatcher et al. [25] also confirmed the presence of miR-430 family in two large clusters of 10 and 57 genes on chromosome 4. The reads which did not show any hits in miRBase were analyzed by BLAST against the Rfam database (ftp.sanger.-ac.uk/pub/databases/Rfam) and Refseq proteins to annotate rRNA, tRNA, snRNA, mRNA and other ncRNA sequences. The maximum numbers of reads were annotated against eukaryotic small subunit ribosomal RNA (SSU_rRNA_eukarya) and tRNA, followed by other ncRNAs (Fig. 4).

### 3.3 Differential expression of known miRNAs

**Fig. 5:**
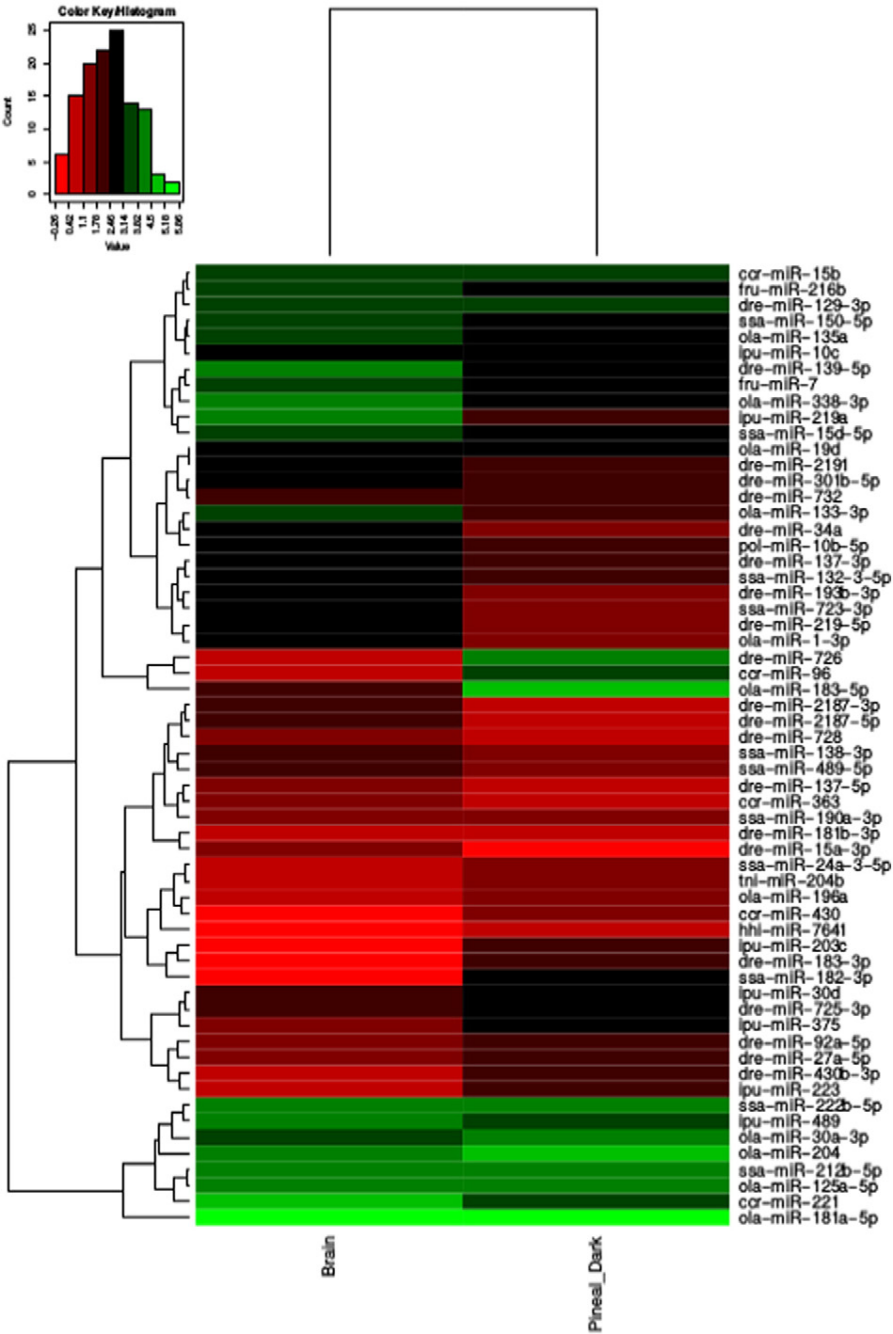
Heatmap of few up-regulated, down-regulated and neutral miRs from the brain and pineal gland.

The differential expression of the miRNA was computed as 2 different sets: 1) brain v/s pineal dark, and 2) brain v/s pineal light. For the brain v/s pineal dark, a total of 93, 99 and 145 miRs were found to be up, down and neutrally regulated, with 49 and 10 miRNAs only expressed in the brain and pineal dark, respectively (Table S2). For the brain v/s pineal light, a total of 105, 101 and 124 miRs were found to be up, down and neutrally regulated, with 56 and 10 miRNAs only expressed in the brain and pineal light, respectively (Table S3). Few up-regulated, down-regulated and neutral miRs from the brain v/s pineal dark are depicted as a heatmap (Fig. 5). The results from both the expression analyses showed that the miR-183/96/182 cluster is highly up-regulated, along with miR-726. The expression of miR-183/96/182 cluster is considerably enriched in the pineal gland and up-regulated by light [23], [24]. These findings are consistent with the light-regulation of this cluster in the mouse retina [26]. This cluster also plays a major role in the regulation of circadian rhythm *via* its targeting of adcy6, a clock-controlled gene that modulates melatonin synthesis [27]. Dre-miR-726 is found to be expressed in the retina of larval and adult zebrafish. From the previous studies it is evident that many miRNAs are transcribed along with their regulating genes, the proximity of miR-726 to SWS2 and LWS opsins suggests that dre-miR-726 could play a vital role in opsin regulation [28].

### 3.4 Identification of novel miRNA candidates

**Fig. 6:**
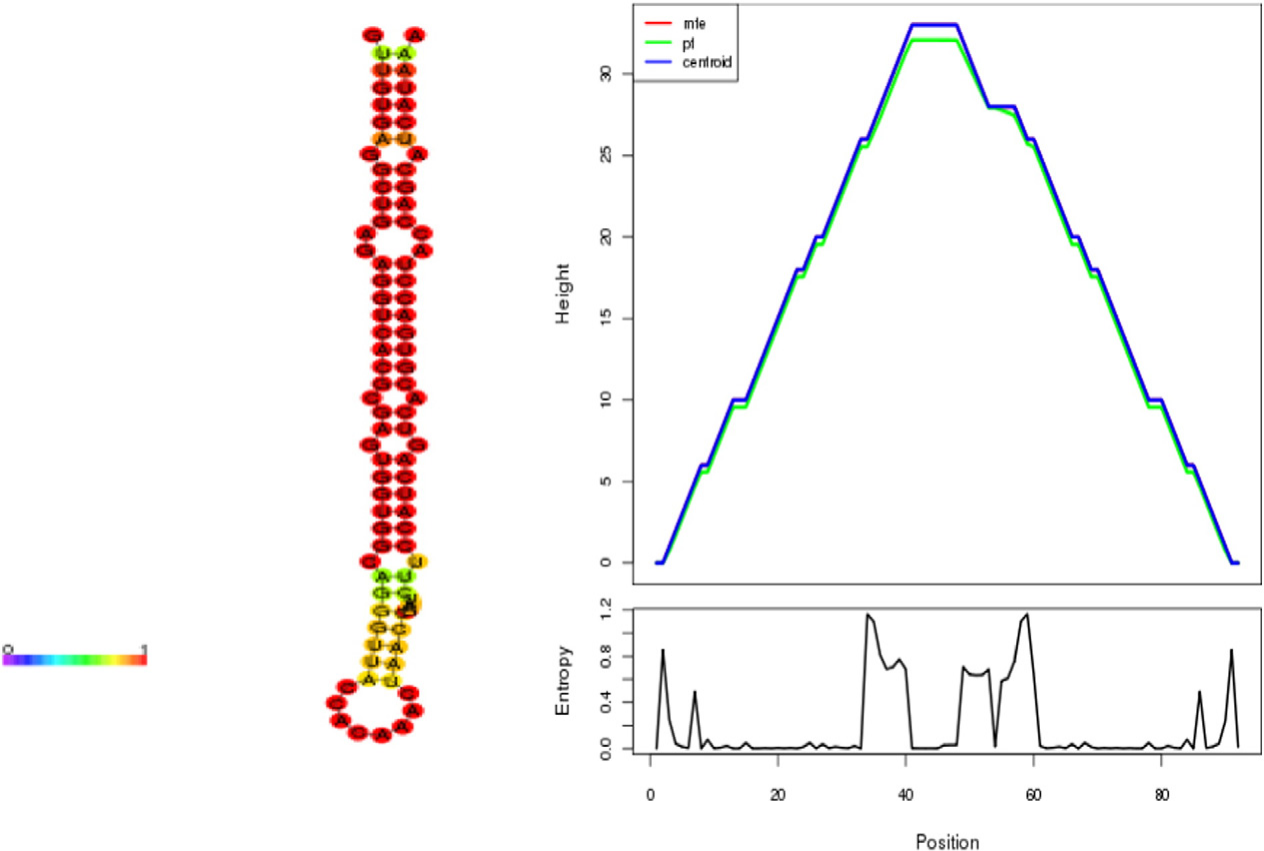
Hairpin structures of Dre-mir-nov74 predicted using RNAfold software.

Mireap was used for the prediction of novel miRs, using reads that did not align to the unique fish miRNA database and Rfam. The reads were aligned onto the zebrafish genome using bowtie (with − v 0). A total of 84.40%, 83.68% and 87.72% of reads aligned for the brain, pineal dark and pineal light, respectively. For a small RNA to be considered as a potential miRNA candidate, it should meet the following strict criteria: 1) the miRNA precursor sequence should fold into an appropriate stemloop hairpin secondary structure, 2) the mature miRNA sits in one arm of the hairpin structure, 3) a maximum of 6 mismatches between the predicted mature miRNA sequence and its opposite miRNA* sequence in the secondary structure, 4) there should be no loop or break in the miRNA or miRNA* sequences, and 5) minimal folding free energy index and negative minimal folding free energy of the predicted secondary structure should be higher. The stem-loop hairpin structures with free energy lower than − 18 kcal/mol as per RNAfold were retained. Mireap predicted a total of 50, 34, and 16 novel miRNAs from the brain, pineal gland (dark treatment) and pineal gland (light treatment), respectively. All of these novel miRNAs were named temporarily in the form of Dre-mir-novnumber, *e.g.* Dre-mir-nov1. To increase the authenticity of the predicted novel miRNA, Zhang et al. [29] combined several parameters to form a new criterion called minimal folding free energy index (MFEI). The MFEI value from all the 3 data sets ranged from − 0.42 to − 1.56. A total of 25, 19 and 9 novel miRNA precursors, with a MFEI greater than 0.85, were identified from the brain, pineal gland (dark treatment) and pineal gland (light treatment), respectively (Table 2). This indicates that the RNA sequences with MFEI greater than 0.85 are most likely to be miRNAs. Most of the novel miRs were also found to originate from chr 4 and chr 5 of zebrafish. The novel miRs ranged from 21 nt to 24 nt in length, with the precursors ranging from 75 nt to 99 nt in length. The free energy of the precursors ranged from − 28.5 to − 71.5 kcal/mol. The secondary structure of a novel hairpin with the highest read count, *i.e.* Dre-mir-nov74, is depicted in Fig. 6. Biological experiments can be undertaken to validate the authenticity of the reported novel miRs.

### 3.5 Target prediction of novel miRs

**Fig. 7:**
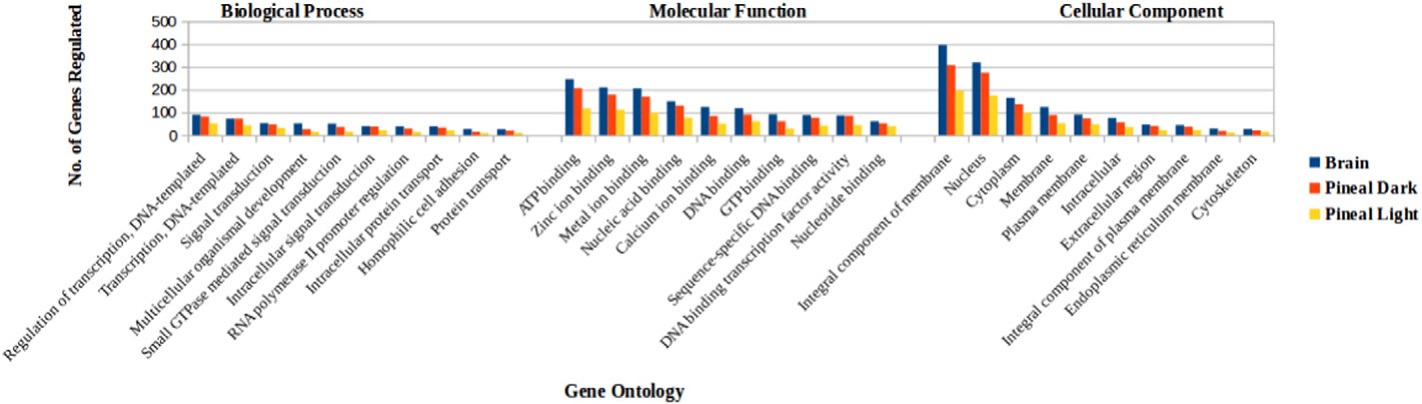
Gene ontology of the target genes of novel miRNAs. The graph represents the maximum represented GO in terms of biological process, molecular function and cellular component.

MicroRNAs bind to the mRNA of the target genes, thus regulating the gene expression at the post-transcriptional level. To gain insight into the function of the newly identified miRNAs, the putative target genes of these miRNAs were predicted using miRanda. The 3′-UTR regions of zebrafish mRNAs were downloaded from ensemble biomart and were checked for their complementarity against the novel miRs. Each miRNA was found to target more than one mRNA. The 25, 19 and 9 novel miRs in the 3 data sets were found to regulate 3737, 2527 and 1719 transcripts (Table S4), with the number of targets ranging from 12 to 873 for each miRNA. In order to infer the functional annotation of the novel miRs, GO analysis was done for the predicted Zebrafish targets, which indicated their involvement in the regulation of diverse physiological processes (Fig. 7). The novel miRNAs were found to play a major role in transcription regulation, signal transduction, organism development, RNA polymerase II promoter regulation, protein transport and homophilic cell adhesion. The pathway analysis also revealed the involvement of these novel miRs in amine and polyamine biosynthesis, carbohydrate degradation, iron-sulfur cluster biosynthesis, urea cycle, AMP and XMP biosynthesis *via de novo* pathway, CTP biosynthesis *via* salvage pathway and tRNA modification. Our study provided further insight into the novel miRNA-mediated regulation of target genes.

## 4 Conclusion

In this study, a total of 25, 19 and 9 novel miRNAs were identified from the brain, pineal gland (dark treatment) and pineal gland (light treatment), respectively, using deep sequencing data and *in silico* bioinformatic analysis. Most of the conserved and novel miRs were found to originate from chr 4 and chr 5 of zebrafish. To gain insight into the function of the newly identified miRNAs, the putative target genes of these miRNAs were predicted using miRanda. Gene ontology analysis was done for the predicted zebrafish targets to infer the functional annotation of the novel miRs, which indicated their involvement in the regulation of diverse physiological processes. The novel miRNAs were found to play a major role in transcription regulation, signal transduction, organism development, RNA polymerase II promoter regulation, protein transport and homophilic cell adhesion. The pathway analysis also revealed the involvement of these novel miRs in amine and polyamine biosyntheses, carbohydrate degradation, iron–sulfur cluster biosynthesis, urea cycle, AMP and XMP biosyntheses *via de novo* pathway, CTP biosynthesis *via* salvage pathway and tRNA modification. A total of 165, 151 and 145 known zebrafish miRNAs were found in the brain, pineal gland (dark treatment) and pineal gland (light treatment) along with their expression values and were further annotated for their presence in other fishes. MiR-181a and miR-182 were the highly expressed miRNAs in the brain and pineal gland of zebrafish. Because of the role of these miRNAs in promoting regeneration and mediating the targets and transcriptional factors involved in regeneration, these miRNAs may prove to be promising in therapeutics. The expression analysis of the known miR genes showed that miR-183/96/182 cluster is highly up-regulated, along with miR-726. The expression of miR-183/96/182 cluster is considerably enriched in the pineal gland and up-regulated by light. The authenticity of the reported novel miRs may further be validated by different biological experiments.

## Competing interests

The authors have declared that no competing interest exists.

## Figures and Tables

**Table 1:** Statistics of the 3 data sets before and after length filtering.

Parameter	Brain	Pineal gland (light treatment)	Pineal gland (dark treatment)
Total number of sequences	14,781,569	5,933,638	10,385,264
Total number of distinct sequences	259,897	265,007	463,789
Total number of sequences after length filtering	14,347,869	5,724,361	10,008,434
Total number of distinct sequences after length filtering	200,520	204,696	351,638

**Table 2:** Zebrafish novel miRNA and their characteristics.

Tissue type	Chromosome	Novel miRNA	Sequence	MiRNA length (nt)	Read count	Precursor position	Strand	Precursor length	% GC	MFEI (− kcal/mol)
Brain	Chr 1	Dre-mir-nov49	AGAGGCUGUCCGAGUGCUGAU	21	156	10303253–10303335	+	83	49.4	− 1.56
Chr 20	Dre-mir-nov8	GACCUGUAACCAUUGACUUCCU	22	102	962031–962117	+	87	36.78	− 1.4
Chr 3	Dre-mir-nov46	AAAAGCGUACCAAACUGAACCGU	23	122	46496327–46496412	+	86	48.84	− 1.3
Chr 17	Dre-mir-nov19	AGACUAGUAGCCAUUGAGAUCUU	23	264	20769194–20769271	−	78	33.33	− 1.27
Chr 18	Dre-mir-nov16	UGUUUUUUUAGGUUUUGAUUUUU	23	1142	45964887–45964967	+	81	33.33	− 1.26
Chr 4	Dre-mir-nov38	GAAUAACUCAAACCGGAGGACU	22	106	31535442–31535523	+	82	47.56	− 1.26
Chr 4	Dre-mir-nov42	AAUAACUCAAACCAGAGGACU	21	126	54513045–54513126	+	82	43.9	− 1.19
Chr 18	Dre-mir-nov15	UUUCCAGAAAGGUCUGUAUGUGU	23	130	26647915–26647993	+	79	46.84	− 1.16
GRCZ10_NA262	Dre-mir-nov2	AGACUCUCCAGUACACUGGCCCU	23	862	744–825	−	82	58.54	− 1.16
Chr 10	Dre-mir-nov25	GAAAACCUGUAACCAUUGACUUCU	24	221	29746032–29746122	+	91	35.16	− 1.14
Chr 20	Dre-mir-nov10	AAACUUGUAAUCACUGACUUCCU	23	125	35014623–35014703	+	81	33.33	− 1.12
Chr 2	Dre-mir-nov48	AAUGACUCAAACCCAAGGACUCG	23	1182	8746947–8747032	+	86	40.7	− 1.1
Chr 6	Dre-mir-nov29	AAACUCUGCAGGACACCAGCUGU	23	268	18113026–18113108	+	83	46.99	− 1.05
Chr 17	Dre-mir-nov18	AAACUCUGCAGGACACCAGCUG	22	748	47039884–47039964	+	81	46.91	− 1.02
Chr 4	Dre-mir-nov39	UAAACUCUGCAGGACACCAGCUG	23	1020	42571226–42571306	+	81	46.91	− 1.02
Chr 6	Dre-mir-nov30	ACUGUACAGACUACUGCCUUGC	22	361	41457248–41457346	+	99	45.45	− 0.98
Chr 18	Dre-mir-nov17	ACUCUUCACUCGUCUGUGUUCA	22	116	50849750–50849835	−	86	55.81	− 0.96
Chr 23	Dre-mir-nov3	UCAAAAGGCGUACCAAACUGUAC	23	155	18099730–18099807	−	78	43.59	− 0.95
Chr 5	Dre-mir-nov37	UCCAUCAGUCACGUGACCUACCA	23	7106	35345784–35345875	−	92	51.09	− 0.94
Chr 5	Dre-mir-nov33	GGCCCGUCCGGUGCGCUCGGAUCC	24	767	824650–824740	−	91	84.62	− 0.93
Chr 5	Dre-mir-nov31	UAAACUCUGCAGGACACCAGCUGU	24	158	10220904–10221001	+	98	46.94	− 0.93
Chr 5	Dre-mir-nov32	GAUGACUCAAACCCAAGGACUCA	23	115	15221789–15221886	+	98	39.8	− 0.89
Chr 8	Dre-mir-nov28	AAUGACUCAAACCCAAGGACUCGU	24	108	2766509–2766596	+	88	44.32	− 0.88
Chr 4	Dre-mir-nov44	AGUGAGGUCCUCGGAUCGGCCC	22	1835	76320181–76320279	+	99	68.69	− 0.87
Chr 22	Dre-mir-nov4	AACGACUCAAGAACCAGAAGACU	23	315	18571986–18572067	+	82	46.34	− 0.86
Pineal gland (dark treatment)	Chr 1	Dre-mir-nov83	AUCAGCACUCGGACAGCCUCUU	22	173	10303251–10303335	+	85	50.59	− 1.53
Chr 17	Dre-mir-nov59	AGACUAGUAGCCAUUGAGAUCUU	23	101	20769194–20769271	−	78	33.33	− 1.27
Chr 18	Dre-mir-nov58	UGUUUUUUUAGGUUUUGAUUUUU	23	381	45964887–45964967	+	81	33.33	− 1.26
Chr 9	Dre-mir-nov66	UACGGGCUGAAUUUAGACAAAUU	23	177	5548615–5548705	+	91	27.47	− 1.25
Chr 24	Dre-mir-nov52	UAACGUUUCGAGCCCACUGACUG	23	118	1825424–1825498	−	75	46.67	− 1.19
GRCZ10_NA262	Dre-mir-nov51	AGACUCUCCAGUACACUGGCCCUC	24	997	743–826	−	84	58.33	− 1.14
Chr 12	Dre-mir-nov62	UUUCCGGAAAGGUCUGUAUGUGC	23	1654	44383412–44383489	+	78	47.44	− 1.14
Chr 4	Dre-mir-nov77	GAAUAACUCAAACCGGAGGACU	22	103	49472235–49472316	+	82	46.34	− 1.11
Chr 6	Dre-mir-nov72	UACGGAUAGAAUCAGCGGAGCGA	23	221	56909252–56909333	+	82	60.98	− 1.11
Chr 8	Dre-mir-nov70	AACCUGUAACCAUUGACUUCCU	22	140	10454696–10454780	+	85	38.82	− 1.04
Chr 1	Dre-mir-nov84	UAAAGCGUACCAAACUGAACCGU	23	150	41154258–41154343	+	86	46.51	− 1.00
Chr 11	Dre-mir-nov63	GCUGAAGUCAUUAUUAUUAGGGC	23	116	5486604–5486685	+	82	35.37	− 0.98
Chr 6	Dre-mir-nov71	ACUGUACAGACUACUGCCUUGC	22	220	41457248–41457346	+	99	45.45	− 0.98
Chr 4	Dre-mir-nov76	GCAGAGAGAAAUGUCUAUGGCUU	23	1712	5403781–5403855	+	75	48.00	− 0.97
Chr 5	Dre-mir-nov74	UCCAUCAGUCACGUGACCUACCA	23	15,880	35345784–35345875	−	92	51.09	− 0.94
Chr 20	Dre-mir-nov54	CUCAUCCCUCUGCUCUAUCCCCU	23	115	37928243–37928325	+	83	50.60	− 0.93
Chr 8	Dre-mir-nov69	AAUGACUCAAACCCAAGGACUCG	23	224	2766510–2766595	+	86	44.19	− 0.90
Chr 12	Dre-mir-nov61	AACGACUCAAGAGCCAGAAGACU	23	323	3216161–3216240	+	80	45.00	− 0.88
Chr 20	Dre-mir-nov55	CAGGGGGUCGGGAAGCACUGCCU	23	4876	48676465–48676553	−	89	58.43	− 0.85
Pineal gland (light treatment)	Chr 1	Dre-mir-nov100	AUCAGCACUCGGACAGCCUCUU	22	100	10303251–10303335	+	85	50.59	− 1.53
Chr 9	Dre-mir-nov91	UACGGGCUGAAUUUAGACAAAUU	23	101	5548615–5548705	+	91	27.47	− 1.25
GRCZ10_NA262	Dre-mir-nov85	AGACUCUCCAGUACACUGGCCCUC	24	491	743–826	−	84	58.33	− 1.14
Chr 12	Dre-mir-nov89	UUUCCGGAAAGGUCUGUAUGUGC	23	1075	44383412–44383489	+	78	47.44	− 1.14
Chr 6	Dre-mir-nov94	UACGGAUAGAAUCAGCGGAGCGA	23	130	56909252–56909333	+	82	60.98	− 1.11
Chr 4	Dre-mir-nov97	GCAGAGAGAAAUGUCUAUGGCUU	23	903	5403781–5403855	+	75	48.00	− 0.97
Chr 5	Dre-mir-nov95	UCCAUCAGUCACGUGACCUACCA	23	8256	35345784–35345875	−	92	51.09	− 0.94
Chr 4	Dre-mir-nov99	AGUGAGGUCCUCGGAUCGGCCCC	23	144	76320181–76320279	+	99	68.69	− 0.87
Chr 20	Dre-mir-nov88	CAGGGGGUCGGGAAGCACUGCCU	23	2104	48676465–48676553	−	89	58.43	− 0.85

## Appendix A Supplementary data

Supplementary Table 1 List of known and conserved miRNA in fishes.

Supplementary Table 2 Differential expression of known miRNAs of the brain and pineal gland (dark treatment).

Supplementary Table 3 Differential expression of known miRNAs of the brain and pineal gland (light treatment).

Supplementary Table 4 List of predicted targets for the novel miRNAs using miRanda.
